# Care of Teeth during Pregnancy and Lactation

**Published:** 1892-02

**Authors:** E. H. Raffenperger

**Affiliations:** Marion, Ohio


### Care of the Teeth During Pregnancy and Lactation.

BY E. H. RAFFENPERGER, D.D.S., MARION, OHIO.

Read before the Ohio State Dental Society, Columbus, Dec., 1891.

This may seem rather a peculiar subject, but it is one that has caused no end of worry and anxiety both to the patient and dentist. It is no longer a mere matter of doubt and speculation that the teeth are seriously affected during this trying period of a woman's life.

In the preparations incident to a wedding now-a-days the dentist is consulted really as often as the dressmaker, and wise is woman who is so thoughtful of the possibilities of the future.

The old saying "a tooth for every child" is only too true, and matters seem to grow worse instead of better, the longer we live.

A woman with poor teeth, is rather the rule than the exception. One half of the married women of to-day will tell us that they had good teeth until the birth of their first child, and that their teeth have been going to ruin ever since.

Whether this destruction of the dental organs at this time is due to the privation of the tooth, of the tooth-building substance, or to an extremely acid condition of the oral secretions, or both, I am not able to say.

Any physician will tell us that his skill has been taxed to the utmost, and his patience sorely tried, in his endeavors to relieve a pregnant woman of some real or imaginary pain and ache in her teeth and jaws. Indeed these pains and aches seem to be a usual accompaniment to pregnancy, nearly as much so as the persistent vomiting, and are about as well understood by the average.physician.

And the skill of the dentist is likewise baffled. For this reason I have chosen this subject, not with a view of displaying my knowledge of it, but with the hope that it may open the way for a thorough discussion in which some of the older and better informed members of this intellectual body can bring out some of the many points which no doubt I have overlooked.

We can all testify that the teeth of a pregnant woman are far more sensitive than otherwise, and that the woman herself is unable to bear the amount of pain, which otherwise would hardly be noticed, and on the whole she is rather an undesirable patient. Still she will call on us, and will continue to do so until the Almighty creates a new order of things; but with a little care and forethought on our part we can save her a vast amount of pain and make for ourselves many friends.

A woman in the early stages of gestation presents herself to us. It does not require a practiced eye to detect her condition; often she will tell us. She desires to have her teeth examined; she is unusually nervous, and her teeth very sensitive, abnormally so; several teeth, say, are found containing cavities. What is to be done with these cavities? Shall we fill them with gold and dismiss our patient, only to have her return in a year or so, with all our work lost? I say no, most emphatically, and I think there are men here who will bear me out when I say it is folly to fill the teeth of a pregnant woman with any idea of permanency.

In the first place she is unable to stand the pain of the preparation of the cavity incident to the introduction of gold, or any of the permanent fillings, and if she were, nature would not assist, as all her attention must now go to the new life that is within and if we can not have the cooperation of this venerable dame, our work is generally for naught.

All we can do, and I am sure it is the best plan, is to fill the cavities with any of the temporary fillings. By these I mean the plastics, which do not require that the cavities shall be so perfectly prepared as for any of the metalic fillings. These temporary fillings are inserted with very little excavating and pain, at the same time answering the same purpose as the gold. We can dismiss our patient for the present and after her child is born and weaned she can have her teeth filled permanently.

She may tell us that there is a sour and bitter taste in the mouth all the time and her teeth are "on edge," and that the act of brushing the teeth is attended with pain, the necks of the teeth being especially sensitive.

These conditions are due to the acid character of the secretions, and can be remedied and largely avoided by the free use of lime water as a mouth wash, and of prepared chalk—this latter most valuable adjunct to the dentist's drug case being packed around the teeth several times a day.

The entire system seems to cry out for calcareous substances, and women will tell of their morbid craving for such substances.

The exquisite sensitiveness found at the necks of the teeth can be governed and cured if the chalk fails, by a little nitrate of silver, pulverised, and applied by means of a sharpened stick first moistened in carbolic acid.

This same remedy, or application, can be used with success on the "canker" sores so often found in mouths of our patients.

As to the extraction of teeth for pregnant women much has been said; some will tell of the dire results therefrom—that it is safer to allow the woman to suffer the "torture of the damned" than to attempt to extract any teeth for her. I contend that if there are any teeth to come out they ought to be extracted at once, as the momentary pain of the operation is not so trying as the continual aching.

I have extracted teeth for women at all stages of gestations and have not heard of any bad results.

One writer sums the whole matter in very few words: he says, "that if the extraction of a tooth would produce miscarriage our offices would be filled all the time with women anxious to have a tooth extracted."

As to the prenatal influences on the child I have never heard of a child being "marked" with a pair of dental forceps or dental engine. So I do not think we have any thing to fear from that source. But I do most firmly believe that when a woman's nervous system is broken down with the toothache the nervous system of her child will also be seriously affected.

We should instruct our patients in the use of such food as will go to the formation of tooth and bone substance or structure. What the child-bearing women of to-day lack is the good, old-fashioned food of our grandmothers, well cooked and plenty of it, such as corn bread, bran bread, mush and milk, etc.

I have frequently had women tell me that their grandmothers lived to a ripe old age and did not have a decayed tooth in their mouths.

The manner of having children has not changed, but the mode of living has made rapid progress toward the worse.

The same care should be given to the teeth during lactation, as there is the same drain on the system.

DISCUSSION.

*Dr. H. A. Smith*, Cincinnati.—I fully agree with the essayist that this is an important subject. But if true, as stated, that the teeth of pregnant women are predisposed to decay, we certainly should be able to give good and intelligent reasons for it. It is supposed that during gestation the structure of a tooth changes from its normality. The statement that there is interstitial change in the relation of the matrix or organic portion of the tooth and lime salts; that the lime salts are perhaps diminished in quantity, has not been proven. If this were true, would it account for the beginning of caries during the period of pregnancy? Caries begins upon the enamel of a tooth. Could the enamel, where we have such a large proportion of inorganic material, be changed interstitially in the period of pregnancy? If it is not true that we have either a chemical or physical change in enamel or dentine during pregnancy, we must look for an active cause of caries in some other direction. It must be due, then, to the character of the secretions of the mouth. If caries, as we believe, begins by a fermentation whereby an acid is formed, then the secretions must be of a nature to promote fermentation in the oral cavity. Does caries attack sound teeth more frequently in pregnant women than in those not in that state? We are not quite prepared to answer that. But if teeth have been filled in the mouth prior to pregnancy and this condition supervenes, I think it possible that we have a recurrence of caries about these operations more frequently than we would have in the absence of this condition. That, of course, must be accounted for on the supposition that the secretions are not normal, that they invite those conditions which induce caries.

*Dr. J. Taft*, Cincinnati.—I am not prepared to controvert the common opinion in regard to this subject. We very frequently, almost universally, hear it said that the teeth during the time of gestation are more susceptible to the influence of decay than they were before that period. Then after that period has passed they return to their original or more normal condition. I do not mean that this is an abnormal condition, but they return to a more favorable condition. My impression is, and I think the experience of every close observer will warrant the conclusion, that the teeth during the time of gestation and lactation do decay more readily than they did before. As Dr. Smith says, it becomes us to ascertain the cause, if possible, of this condition. I take it that this is found in perhaps two or three things. We are brought to this conclusion by observation; though not as many experiments have been made in this line as there might have been. The analyses during both these different periods of life might easily be made by some one who would take it up and carry it on and determine some of the facts to which reference has been made, especially with regard to the change in the relative amount of constituents of the dentine. I have no doubt there is a change in the relative amount of the organic and inorganic constituents of teeth. That expression is subject to criticism, but we all know what it means, and with it we may rest satisfied for the present, at least. I do not see how there can be any structural change in the tissue. There may be a change in the relative amount of the organic and inorganic material by one or the other being withheld. Then there would be a change in the relative amount of these materials.

It has been assumed by many, and the opinion is largely entertained, that in the demand upon the nutrient function of the body the inorganic material of the teeth is not as liberally supplied during the period of gestation and lactation as before. It seems a rational thing, and perhaps it is supported somewhat by analogies which we might trace as well. I am hardly able to explain the phenomena which perhaps every close observer has realized in this particular. It is often found that teeth prior to this period are firm and hard and not predisposed to decay; perhaps not decayed at all, but soon after or almost cotemporaneous with the occurrence of gestation the teeth begin to decay. They are also more sensitive. Observation teaches us that during this period more than at other times the teeth are likely to decay, or to take on a higher degree of sensitiveness than they did before, clearly indicating that some change had taken place. Is that dependent upon a change in the relative amount of the constituents of the tissues, or may it depend upon something else? It is clear that there is a change. Some teeth are more resistant, are more hard and dense, and but little disposed to decay. One of the explanations of this is in the change of material that is found in connection with the disturbance of nutrient function, and causes also an excited nervous irritability in the tissues and in the body as a whole, because there is a higher state of nervous excitability under this condition than prior to it or after it has passed by. Another is a vitiation of the oral secretions; a vitiation not only of the oral secretions, but of the gastric secretions as well. The nausea of this period indicates much. This nausea does not occur without some gastric disturbance, and the gastric secretions and the secretions of the mouth will also be influenced manifestly by the condition that brings about the nausea. The secretions of the mouth are not only vitiated, as can evidently be ascertained by analysis and close examination, but the gastric juices are also vitiated. Indigestion accompanies this condition of nausea which is very unfavorable. It is unfavorable to the proper performance of the nutrient functions. If there is indigestion, and there usually is, there is impairment of the process of nutrition, and not only will one or two tissues of the body suffer, but all that receive nutrition will suffer as well. The teeth, therefore, will suffer on this account from a want of nutrient supply. It has been stated that there is a demand made upon the nutrient function of the body for an increased nutrient supply during gestation, and that in proportion as this new demand is made, the tissues of the mother will fail in receiving their proper supply. I have no doubt this will occur in cases where the digestion and the assimilating power are not strong, where there is not proper exercise of the body. There is, no doubt, cases in which this function is so well performed that there is no general impairment experienced anywhere in the system, but in those cases enfeebled in any way, or not strong, they would necessarily suffer, and especially so if these interruptions in the digestive and nutrient processes were set up. Under these circumstances there will be more or less a deficient supply in the nutrition of the various tissues of the body and of the teeth as well. It may be asked, Why don't other tissues of the body suffer as well as the teeth? If closer attention were given, this would be found to be true. It is true in many cases that there is a high degree of nervous irritability which exists then and does not exist under other circumstances, or did not exist before. If this be true, to what are we led in the way of a remedy? We should simply avoid those things that would disturb or impair proper function. If there is enfeeblement in any wise, tone up, strengthen, support the system and aid the nutrient processes, so that a sufficient supply may be made to all the structures of the body of both the mother and the fœtus as well. Also protect the mother during this time as perfectly as possible from all irritation, from all disturbance both mental and physical, and have her maintain the best possible surroundings and conditions for the carrying on of that great and important function that is being carried on in her body. This is a great and important matter, and great care ought to be exercised for the mother during the time of gestation and lactation as well, because some impairment is as likely to occur during the period of lactation as during that of gestation. Careful attention is needed here in the way of giving proper food, having the system in the best possible condition for digesting and assimilating it. We should have the mental condition of the mother in as perfect a condition as possible, free from all exciting and disturbing influences, free from grief and over-joy, free from any perturbations or excitements of the mind that would tend to destroy the equilibrium or the balance of the various functions of the body. When that is done, about all is done that can be, except the local treatment which was mentioned in the paper. This is a mere surface matter. The matter of keeping the mouth in as healthy condition as possible by local treatment, the matter of attention to the teeth, relieving them from great disturbance from aching or from diseases, and filling the cavities of decay with the least possible disturbance to the patient, are all clearly indicated. These things constitute the principal treatment during the time of gestation and lactation.

*Dr. A. O. Rawls*, Kentucky.—I do not believe I can contribute any thing of interest to this subject. There is very little to be said beyond what we have heard advanced by authorities on kindred subjects. I am somewhat inclined to doubt the theory or statement advanced by Dr. Smith, that there is no difference at any time between the organic and inorganic substances. In the first place it does not seem to me to be consistent, even though I can not prove by the aid of the microscope that there is a difference between organic and inorganic substances. There are a great many things we can not prove that we really know to be so. It is said that there is very little, if any, nutrition between the terminals of the nutrient current as it is known to-day to the microscope, and yet we can not perceive how tissue can be made at those points where, apparently, there is no nutrition or circulation of the nutrient current, and yet it is so. So it seems to me that when there is a disturbance, an aberration of the function of a tooth substance from any cause, whether it be lactation, gestation, or what not, there must be a change in the amount of the assimilation. We could not have disturbances without that. My idea about that is, that the tooth loses to a certain degree, not exactly its assimilating property, but the amount of substance that it formerly had to supply the waste particles as they are passed off. You say there are no absorbent vessels in the dentinal tubules; nevertheless there is nutrition, and I have believed of late years that there is a disturbance of the nutrient supply to all parts of that tooth, both the dentine or the enamel. Now if there be that condition, and if the enamel itself is taken into the nutrient circulation, if that current of nutrition has been at all interfered with by any process, we are liable to have a weakened condition in the tooth substance, and there is liable to be a deficiency of the chemical constituents of the inorganic or organic material, otherwise we could not have this want of nutrition where the microscope fails to find the current, the canals or the directions by which the nutrient supply goes to the different parts. Wherever we fail to find out that, I believe beyond there are those canals or some means by which this nutrient supply is kept up which the microscope can not see. It does not seem to me to be reasonable to believe any thing else. I believe that this current is kept up between the enamel of the tooth and the end of the current. I believe there is a difference between the aged and youth, and between the periods of gestation, lactation, and other periods in the life of a woman, that makes it more easy for any acid or alkaline condition, as the case may be, to affect a tooth. It is true where the enamel is not entirely broken away, if we go above that we might not have decay, yet underneath the enamel we may have a weakened condition of the enamel itself. I believe that within the substance of the enamel there is just as much life in it comparatively as there is in any other part of the body. I do not believe we have a thin line around us, or a thin skin without more or less life in it. We all know that the skin appendages must have nutrient supply to keep them in action, otherwise they come off the same as the condition in catarrhal inflammation. They are all thrown off, they can not exist in different parts of the body, they must have life. My finger-nails have life in them. They must have nutrition or they would not grow; therefore, while I do not believe there is a disturbance from these conditions, there is, I think, a lessening of the nutrient supply from some source by some reason or other to the tooth of the mineral constituents. We know that in the plastidule the earthy matters are grown to form a higher type of life. We know that the higher the type grows the more lime salts it would need; so it is with the fœtus in the mother's womb. It is a large plastidule, it has no mineral substance in its body, and there is a greater demand for that special material on the part of the child than there is for any other material. Therefore the current of nutrition that carries this substance to be assimilated, or rather the demand of that particular portion of the body for lime salts is so much greater that it is taken in large quantities, and probably thus deprives other parts of the body of their original amount of supply. We have no absolute means at our command of telling just exactly how this is brought about, nor is there any special means of proving it. We have to grope more or less in the dark in these cases. We can not reason as well upon this subject as we have on many others which we know just as little about, yet we know to be true according to our clinical knowledge.

As to the means of treatment, that portion of the subject has been touched upon by Drs. Taft and Smith, and the writer of the paper. We forget in our eagerness to be useful to mankind, and especially with women in this condition, that they are not the masters of the situation always. A woman can not be pleasant under these circumstances. She can not have a happy frame of mind, a calm demeanor and good habits at all times, while she is in such a condition if she would, and in nine cases out of ten they can not attend to their daily duties. In some cases they can not take proper care of their children, do their duty and keep their frame of mind which Dr. Taft speaks of. It is an utter impossibility. The mental anxiety attending this condition is considerable, and we can not break in on the natural tendencies or proclivities of the mind of the mother, or the tendencies of physique, when she is in that condition ; in other words, we can not break into those conditions which seem to be natural to that condition of the body.

With regard to the use of local remedies for the treatment, we might possibly help things by advising the use of a supplemental diet, but the question arises whether this diet will be assimilated or not. Nine times out of ten, I am certain, in which you can use all of the means possible for improving the condition of the body, that the patient will be just as well off if they hadn't used local remedies and supplemental diet at all. What is given will not be assimilated or taken up. We want a condition to assimilate the substances that are taken.

*Dr. J. Taft*.—I fully agree with Dr. Rawls in his statement that persons are not always masters of the situation, all I intended was simply to say that the best should be done under the circumstances. Many of us know that much more can be done than is ordinarily accomplished, that prospective mothers are subjected to annoyances unnecessarily, and a great many things can be avoided if care is exercised. That is all I plead for, that the best condition shall be established and maintained. I recognize the periods of excitement and stress of mind that necessarily exist in some cases. With many women these conditions are not present. After all it should be our aim to free such persons from all irritability, from all the embarrassments possible, and then trust to providence for the rest.

In regard to food, we have a lesson in the kind of food that is so anxiously sought many a time by the patient. You know oftentimes that one article or another of food is anxiously desired, and perhaps it is wise that it shall be given or had by the patient. It will be said that this is subject to certain conditions and variations. This is very probable. After all, if the best possible physiological condition is maintained, there will be the least possible embarrassment in connection with it.

*Dr. C. M. Wright*, Cincinnati.—I was very much interested in listening to the paper and the discussion on it. It seems to me that wherever there is life, it must bear some relation to nutrition, and that relation means not only the reception of nutritious material, but the giving off of waste and eliminating certain forces in the way of heat, etc. The relative vitality of a tooth is a question that we cannot discuss at this time. There is one point, however, and that is—a tooth bears a certain likeness to bone, which has been referred to by both Dr. Smith and Dr. Rawls in their remarks. The question is whether the chemical relation between the lime salts—the mineral, and the animal matter, is fixed or not. Some French experimenters claim, after making careful examination and analyses, that they found a certain per cent., let us say approximately of about 30 per cent. of animal and 70 per cent. of mineral matter in normal bone. They fed the animals (pigeons, rabbits, etc.) on food from which the lime salts were carefully excluded. After starvation which followed the exclusion of the proper proportion of lime salts, chemical analyses were made, and the quantities remained the same relatively; therefore they made the statement that it was not a *mixture* of lime salts and animal matter but a chemical union, and that the same results followed starvation from any cause. Removing the nitrogenous substances from the pabulum, and feeding them on non-nitrogenous, substances, the bone substance starved, but the chemical proportions remained the same. This has been studied by Charcot and other French writers. How is it then that young bone is soft and pliable, and old bone brittle? Has not old bone more lime salts in it than young bone? No; it is because of the more active life and greater nutritive capacity of young bone. It is more active in taking up nutriment and eliminating waste, and not because it has less lime salts, that we find that it knits more readily after fracture.

I was so interested in this subject that years ago I wrote to Dr. Miller, of Berlin, asking him why a tooth this year could be soft and next year hard, and whether it was due to a change in the relative amount of lime salts?

Two or three years ago Dr. Kirk made the statement (he had control in some way of the diet at a Children's Hospital), which I give loosely, that children upon entering the hospital were in such a condition that he could cut away the dentine of their teeth very easily. They followed a certain diet for a year and the teeth became flinty, so that fire would fly from the excavator. The inference was that this food containing lime furnished a greater supply of lime salts, which was taken up by the living matter of the teeth. Clinically we *feel* a difference with our excavators between soft and hard teeth. We have touched teeth that have seemed soft and not to have had a very large supply of lime salts, while at other times we have touched them and found the opposite condition. The theory advanced seems to be against our clinical knowledge. I am in the habit of teaching nursing mothers that by supplying them freely with lime salts, their children's teeth will improve in quality. It seems plausible though I am not certain that it is true according to theory. This subject is of considerable interest, but we have no positive reasons why we do thus and so. It seems to me that we do successful things in an entirely empirical way sometimes.

*Dr. C. R. Butler*, Cleveland, — I am glad Prof. Wright referred to the condition of the bones and tissues of the child and of those of the person more advanced in age. It is a recognized fact that in the child, not only so far as the osseous structure is concerned, but in the whole make-up, there is an activity and elasticity which we do not find in the aged. It is not because there is a greater amount of the lime salts in the bones of the aged than in the child, but that the vital forces are carried on in such equipoise. The nutrition, waste, and repair go on so rapidly that activity and elasticity follow. This is not the case in the aged. It is a recognized fact in physiology that the blood vessels become stiffened and even calcific matter is found present which results in destruction. We do not find that so in the child, because there is vital power enough to keep the flow going on. We have cases where the skin and peripheral surface become, as we say, dead, because the current does not flow around these little points; there is a lodgement of the waste material; it is not eliminated, but lodged on the peripheral surface, therefore we get what we commonly term a "blocking-up."

He speaks of the theory or supposition we have been laboring under; that the teeth do become solidified or calcified in the young subject; that they are easily cut. I recall the statements made by Dr. Kirk, that clinically we know that the young subject's teeth seem to cut away very readily, while after a few years—say four or five years—they are very much harder. Have we really been working in the right direction, or have we the right conception of what causes these different conditions? There are certain conditions during the period of gestation and lactation which are commonly observed, but just what causes them I am not able to state, nor am I satisfied with the statements that have been made with reference to them.

When I saw this paper on the program I was in hopes it would bring out some new features. There is a paper which has been prepared for another society on the same subject by a gentleman in Cleveland which will probably be read next week. I hope he will have something new or better to offer in this direction than has been heretofore.

*Dr. C. H. Harroun*, Toledo.—There is one point which has not been spoken of either in the paper or discussion, and it is that which relates to the extraction of the teeth of pregnant women. Does it produce bad results? Physicians either actually give the lie to what they believe, or else they tell the truth. They often come to me and request me not to extract teeth for patients that are pregnant, for fear of producing a miscarriage. I do not believe in that theory. When such patients have presented themselves to me I have extracted their teeth without any ill-results. I have never known a case to be followed by unfavorable results, and I have been practicing for thirty years. Do we have marks or impressions produced upon the infant through operating upon the teeth of the mother? That is a question which physicians look to a great deal. Our own mothers believe it. We see disfigurements of the child after it is born, and the mother declares that violence was produced at a certain time.

I removed quite a number of teeth from a lady whom I did not know was pregnant. She was quite a strong, stout woman. I noticed one thing which attracted my attention. Every time I proceeded to extract a tooth for her, she would throw herself back as if in a spasm. Sometime afterwards the midwife came to me and said, "Do you remember extracting some teeth for a certain lady?" Yes, said I. "Well, she has given birth to a dead child, and it is a good thing it was still born." Why? "Because its little mouth was torn to pieces, and its mother stated at the time she had the teeth extracted that it had a tremendous effect upon her." She became restless, could not sit still, and she threw herself into a violent spasm every time I attempted to extract a tooth. I did not disfigure the mouth at all. The roots of the teeth that were broken off were removed, leaving the mouth in a good condition.

The following three papers read by Drs. C. M. Wright, H. H. Harrisson, and A. O. Rawles, the one directly following the other; the subjects treated by each having a very near relationship, discussion upon the three papers was had at the conclusion of the reading, for which see subsequent page.

